# *Balanophora spicata* and Lupeol Acetate Possess Antinociceptive and Anti-Inflammatory Activities *In Vivo* and *In Vitro*

**DOI:** 10.1155/2012/371273

**Published:** 2012-11-11

**Authors:** Yuh-Fung Chen, Chien Ching, Tian-Shung Wu, Chi-Rei Wu, Wen-Tsong Hsieh, Huei-Yann Tsai

**Affiliations:** ^1^Department of Pharmacology, China Medical University, No. 91 Hsueh-Shih Road, Taichung 40402, Taiwan; ^2^Department of Pharmacy, China Medical University Hospital, Taichung 40427, Taiwan; ^3^College of Pharmacy, China Medical University, Taichung 40402, Taiwan; ^4^Chinese Medicine Research and Development Center, China Medical University Hospital, Taichung 40427, Taiwan; ^5^Department of Chemistry, National Cheng Kung University, Tainan 70101, Taiwan; ^6^School of Chinese Pharmaceutical Sciences and Chinese Medicine Resources, China Medical University, Taichung 40402, Taiwan

## Abstract

Aims of the present study were to investigate effects of *Balanophora spicata* (BS) on antinociception and anti-inflammation both *in vivo* and *in vitro*. Crude extract of BS inhibited vascular permeability induced by histamine, serotonin, bradykinin, and PGE_2_, but not by PAF. Furthermore, BS crude extract, different layers (*n*-hexane, ethyl acetate, *n*-butanol, and water layer), and lupeol acetate had significant antinociceptive and anti-inflammatory effects on acetic acid-induced abdominal writhing response, formalin-induced licking behavior, carrageenan-, and serotonin-induced paw edema. The *n*-hexane layer had the most effective potency among all layers (IC50: 67.33 mg/kg on writhing response; IC50s: 34.2 mg/kg and 21.29 mg/kg on the early phase and late phase of formalin test, resp.). Additionally, lupeol acetate which was isolated from the *n*-hexane layer of BS effectively inhibited the acetic acid-induced writhing response (IC50: 28.32 mg/kg), formalin-induced licking behavior (IC50: 20.95 mg/kg), NO production (IC50: 4.102 *μ*M), iNOS expression (IC50: 5.35 *μ*M), and COX2 expression (IC50: 5.13 *μ*M) in LPS-stimulated RAW 264.7 cells. In conclusion, BS has antinociceptive and anti-inflammatory effects which may be partially due to the inhibition of changes in vascular permeability induced by histamine, serotonin, bradykinin, and PGE_2_ and the attenuation of iNOS and COX-2 expression.

## 1. Introduction

*Balanophora spicata *Hayata (BS) is a native parasitic plant that grows in rhizome or roots of various hosts in Taiwan. BS is used in daily life as a folk medicine for various treatments including pyresis, algesia, and inflammation [1]. Some *Balanophora* genus plants contain triterpenoid compounds, such as *β*-amyrin palmitate (balanophorin A), lupeol palmitate (balanophorin B), *β*-amyrin acetate, lupeol acetate, *β*-amyrone, leupone, and palmitic acid [2]. The whole plant of BS contains balanophorin A, balanophorin B, *β*-amyrin acetate, monogynol, leupone, caffeic acid ethyl ester, catechin, and 1-O-(E)-caffeoryl-3-O-gallucopyranose [3]. Phenolic constituents from BS possess DPPH radical scavenging activity [4]. Recent published paper found that isolariciresinol, the anti-inflammatory compound from *Balanophora laxiflora*, reduced NO production in LPS-treated RAW 264.7 cells [5]. However, there are no reports on the antinociceptive and anti-inflammatory activities of BS *in vivo*. In the present study, the skin window test was used to evaluate the anti-inflammatory effects of BS crude extract on changes in vascular permeability caused by inflammatory mediators, such as, serotonin, histamine, bradykinin [6], platelet activating factor (PAF), and prostaglandin E_2_ [7–10]. The antinociceptive and anti-inflammatory activities of BS were studied by using an acetic acid-induced writhing response [11], formalin-induced licking behavior [12], and carrageenan-, serotonin-induced hind-paw edema test [13–15]. In addition, the effects of lupeol acetate on LPS-stimulated NO production, iNOS, and COX_2_ expression in RAW 264.7 were also used to evaluate the anti-inflammatory mechanism of BS. 

## 2. Materials and Methods

### 2.1. Plant Collection and Identification

**Figure 1:**
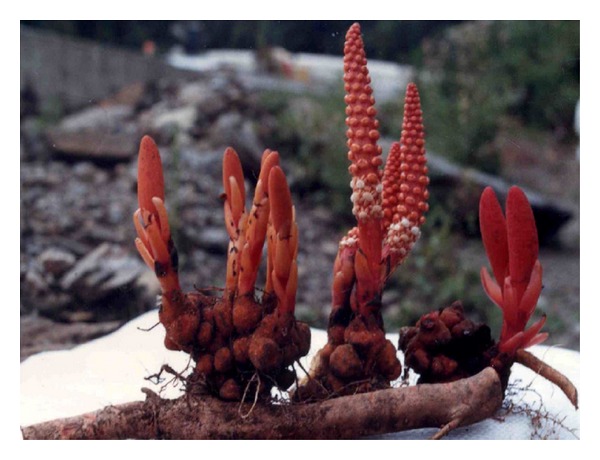
*Balanophora spicata* Hayata was sampled from the Meifeng farm at Ren-ai Township, Nantou County, Taiwan.

BS was sampled from the Meifeng farm at Ren-ai Township, Nantou County, Taiwan (Figure 1) and a plant sample was deposited in the herbarium at China Medical University (no. ICPS-B2000001), Taichung, Taiwan.

### 2.2. Extraction, Isolation of Plant Extract

**Figure 2:**
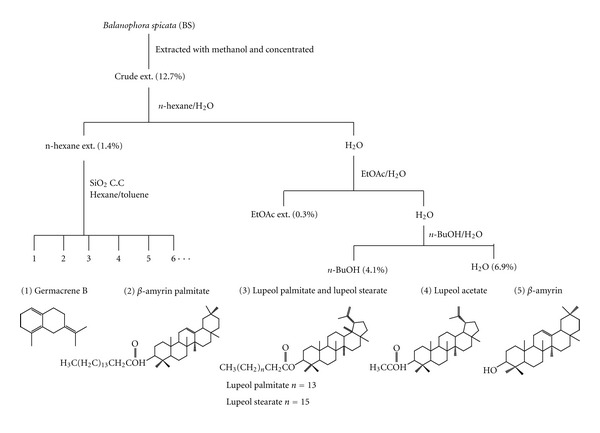
Top: A flowchart of the separated method procedure for isolation of active component from *Balanophora spicata *(BS). Bottom: the structures of five compounds isolated from the *n*-hexane-layer of BS, germacrene B (1), *β*-amyrin palmitate (2), lupeol palmitate and lupeol stearate (3), lupeol acetate (4), and *β*-amyrin (5).

A fresh sample of BS was chopped into small pieces and extracted with methanol 5 times. The resultant extracts were combined and concentrated under reduced pressure to obtain the crude extract of BS, whose yield rate was 12.7%. The crude extract then was dissolved in water and was partitioned successively with *n*-hexane, ethyl acetate, and water-saturated *n*-butanol to obtain four layers in yield rate of 1.4%, 0.3%, and 4.1%, and the yield rate of the aqueous layer was 6.9%. The *n*-hexane layer was evaporated to dryness and applied to a silica gel column and eluted successively with *n*-hexane, toluene, and ethyl acetate. Five compounds obtained from the *n*-hexane layer of BS were germacrene B (1), *β*-amyrin palmitate (2), lupeol palmitate and lupeol stearate (3), lupeol acetate (4), and *β*-amyrin (5) (Figure 2). All of the known triterpenes were identified by comparison with authentic samples by their IR, MS, NMR spectra data. Among those five compounds, lupeol acetate was the most abundant one, whereas, germacrene B was found with a very low yield as compared to lupeol acetate under the same experimental conditions. Therefore, in the present study we mainly attempted to explore the antinociceptive and anti-inflammatory activities of crude extract, *n*-hexane layer, ethyl acetate layer, *n*-butanol layer, water layer of BS, and lupeol acetate *in vivo* and *in vitro*.

### 2.3. Chemicals

 Carboxymethylcellulose, acetylsalicylic acid (ASA), indomethacin (INDOL), carrageenan, serotonin, histamine, bradykinin, platelet activating factor (PAF), prostaglandin E_2_, Evans blue, sodium nitrite, Griess reagent, lipopolysaccharide (LPS), and anti-*β*-actin antibody were purchased from Sigma-Aldrich (St. Louis, MO, USA). Dulbecco's Modified Eagle Medium (DMEM), glutamine, and fetal bovine serum (FBS) were purchased from Gibco (Invitrogen, Grand Island, NY, USA). Anti-iNOS and anti-COX2 antibodies were purchased from Abcam (Cambridge Science Park, Cambridge, UK).

### 2.4. Preparation of Plant Extracts and Drug

Crude extract, each layer of BS, and lupeol acetate were dissolved in 0.5% carboxymethylcellulose (CMC) and intraperitoneally administered 30 min prior to the algesic (acetic acid or formalin) or inflammatory agent (carrageenan, serotonin, histamine, platelet activating factor, or prostaglandin E_2_) injection. Acetylsalicylic acid (ASA, 150 mg/kg) and indomethacin (INDOL, 4 mg/kg) as positive control were prepared as suspensions with 0.5% CMC and administered intraperitoneally 30 min prior to the injection of the inducer (such as acetic acid, formalin, carrageenan, serotonin, histamine, bradykinin, platelet activating factor, and prostaglandin E_2_). The inducer was dissolved in normal saline and used in nociceptive behavior, hind-paw edema test, and in the vascular extravasation test.

### 2.5. Animals

Male ICR mice, weighing 20–25 g, were used for the antinociceptive activity. Male Wistar rats, weighing 200–250 g, were used for the vascular permeability and anti-inflammatory experiments. Animals were obtained from the Laboratory Animal Center, National Taiwan University (Taipei, Taiwan). All animals received humane care, and the study protocol was approved by the Institutional Animal Care and Use Committee (IACUC), China Medical University, under the code 100–223. The animals were housed for at least one week before starting the experiments with free access to standard food pellets (supplied and designed by Fwusow Industry Co. LTD., Taiwan) and tap water. Animals were kept in a regulated environment (23 ± 1°C temperature and 60% humidity) on a 12–12 hr light/dark cycle (light phase: 08:00–20:00 hr). After behavioral measurement, all animals were euthanized with carbon dioxide. 

### 2.6. Skin Window Test in Rats

This microvascular permeability was determined in rats by measuring the absorbance change in the abdominal Evan's blue extravasations after the intradermal injection of inflammatory mediators such as serotonin, histamine, bradykinin, PAF, and PGE_2_. Crude extract of BS (50, 100, 250 mg/kg) was administered intraperitoneally to rats 30 min prior to intradermal injection of inflammatory mediators. Thirty minutes after BS treatment, rats were anesthetized with 30 mg/kg pentobarbital, and then shaved their abdominal hairs and marked with six 2 cm diameter circles in abdominal skin [16]. Two minutes after the intravenous injection of 50 mg/kg Evan's blue, the animals were intradermally injected with 50 *μ*L saline, serotonin (1 nM), histamine (10 *μ*M), bradykinin (10 nM), PAF (40 nM), or prostaglandin E_2_ (2 nM) into the central area of six circles in the abdominal skin, respectively. After 1 hr, rats were sacrificed, and the stained skin at the injected site was excised. The stained skins were infiltrated with 3 mL 0.5% sodium sulfate and 7 mL acetone overnight to extract the abdominal Evan's blue extravasations. The infiltrated solution was centrifuged at 2000 × g for 20 min, and the supernatant was collected and absorbance was measured at 590 nm [9]. The alternation of vascular permeability was obtained for each group by using the following ratio: (*A*_induced_ − *A*_saline_)/*A*_saline_∗100, where *A*_induced_  is the absorbance of Evan's blue extravasations in the circle treated with inflammatory mediator, and  *A*_saline_  is the absorbance of Evan's blue extravasations in the circle treated with saline.

### 2.7. Effects of BS on Inflammation in Rats

The anti-inflammatory activities of BS were determined in rats by measuring the mean increase in hind paw volume after a subplantar injection of inflammatory agent such as carrageenan or serotonin [13–15]. One percent of carrageenan or serotonin was injected in the right hind foot under the plantar aponeurosis. Inflammation was quantitated in terms of milliliters using a plethysmometer (7150 Ugo Basile), which recorded small differences in water level caused by volume displacement. Before any treatment, the average volume of the hind paws of each animal (Vo) was determined with three measurements that did not differ by more than 4% (preciseness of the apparatus). Then 30, 60, 90, 120, 180, 240, 300 min after the injection of the inflammatory agents, the average volume of the hind paws of each rat (Vt) was determined with three measurements that did not differ by more than 4%. The percentage of edema at each record was calculated by comparing the average volume of the hind paws of each rat after the injection of the inflammatory agents with the average volume of the hind paws of each rat (Vo) before any treatment. Percentages of inhibition obtained for each group by using the following ratio: [(Vt/Vo) control − (Vt/Vo) treated]/(Vt/Vo) control ∗ 100.

Rats were pretreated with BS crude extract (50, 100, 250 mg/kg, i.p.) 30 min prior to a carrageenan or serotonin injection. In order to explore the active layer of BS effects on carrageenan-induced paw edema, BS each layer (100 mg/kg, i.p.) was administered 30 min prior to carrageenan injection. Lupeol acetate (100 mg/kg, i.p.), isolated from *n*-hexane layer of BS, was administered 30 min prior to carrageenan injection to further study its anti-inflammatory effect. Indomethacin (4 mg/kg, i.p.) was used as a positive control.

### 2.8. Effect of BS on Acetic Acid-Induced Abdominal Writhing Response

Mice were intraperitoneally administered with crude extract various BS layers or lupeol acetate (10, 25, 50 mg/kg), acetylsalicylic acid (150 mg/kg), or indomethacin (4 mg/kg) 30 min before testing. Control animals received an equivalent volume of vehicle solution. Then each mouse was given 1% acetic acid (10 mL/kg, i.p.). Injection of acetic acid (1.0%) results in contraction of the abdominal muscle together with a stretching of the hind limbs [6]. After the challenge, mice were individually placed into plastic cylinders of (20 cm in diameter) and the numbers of writhes were recorded for 10 min. The number of writhes permitted us to express the percentage of protection using the following ratio: (control mean − treated mean)/control mean ∗ 100.

### 2.9. Effects of BS on Formalin-Induced Licking Behavior

The procedure used for the formalin-induced licking behavior was similar to one described previously by Tsai et al. [17] and Shibata et al. [12]. Mice were pretreated with crude extract, various layers of BS, lupeol acetate (10, 25, 50 mg/kg, i.p.), or indomethacin (4 mg/kg, i.p.) 30 min prior to a 1% formalin injection. Animals were then placed in an observation chamber on an acrylic transparent plate floor 5 min prior to the formalin injection. In order to allow clear observation of the animal's paws, a large mirror was inclined at a 45° angle beneath the floor [18]. After the formalin injection into the right subplantar of the mice, mice were returned to the chamber and observed for two distinct periods of a licking response. The first period (early phase) was recorded 0–5 min and the second period (late phase) was recorded 10–35 min after the formalin injection. The time(s) spent licking the injected paw was measured as an indicator of pain response.

### 2.10. Cells

 RAW 264.7, a mouse macrophage cell line, was obtained from the Bioresource Collection and Research Center (BCRC, Hsinchu, Taiwan), and used for the study of lipopolysaccharide- (LPS-) stimulated NO production, iNOS and COX2 protein expression. Cells were cultured in Dulbecco's Modified Eagle Medium (DMEM; Gibco/BRL) supplemented with 4 mM glutamine and 10% fetal bovine serum (FBS; Gibco/BRL) and maintained in a 37°C humidified incubator containing 5% CO_2_. 

### 2.11. Effect of Lupeol Acetate on LPS-Stimulated NO Production in RAW 264.7 Cells

RAW 264.7 cells were seeded in 96-well plates (5 × 10^4^ cells/well) supplemented with DMEM and 10% FBS and were incubated for 12 hr. Cells were incubated with different concentrations of lupeol acetate (2, 4, 8 *μ*M) for 2 hr. Then, RAW 264.7 cells were treated with 0.1 *μ*g/mL LPS, and incubated for 24 hr, and a control group was treated with LPS only. Nitrite production was determined using the Griess reagent protocol. At room temperature, incubated 100 *μ*L of medium with an equal volume of Griess reagent for 15 min, and nitrite production was quantified using a microplate reader at 540 nm. 

### 2.12. Effect of Lupeol Acetate on LPS-Stimulated iNOS and COX_2_ Protein Expression in RAW 264.7 Cells

Sample proteins (50 *μ*g) were electrophoresed by using 10% sodium dodecyl sulfate-polyacrylamide gel electrophoresis (SDS-PAGE), and then transferred to a nitrocellulose membrane. Anti-iNOS and anti-COX2 antibodies (Abcam, Cambridge Science Park, Cambridge, UK) were used for the iNOS and COX2 expression assay, and enhanced chemiluminescence detection kit was used for the immunodetection (Amersham, Piscataway, NJ, USA). Each experiment was conducted at a minimum in triplicate.

### 2.13. Statistical Analysis

Data were expressed as mean ± standard error (SE) and analyzed using one-way analysis of variance (ANOVA), followed by Scheff's test. When the probability (*P*) was less than 0.05, the difference was considered to be significant.

## 3. Results

### 3.1. Chemical Composition of BS

 Five compounds were obtained from the *n*-hexane layer of BS including germacrene B,  *β*-amyrin palmitate, lupeol palmitate/lupeol stearate, lupeol acetate, and *β*-amyrin which were identified by IR, MS, and NMR (Figure 2). All of the known compounds were identified by comparison with authentic samples by their NMR or MS spectra data. Germacrene B. C_15_H_24_, M204. The IR*ν*_max⁡_^KBr^ cm^−1^: 2900, 1640, 1440, 1370, 1150, 1080. EIMS *m*/*z* (rel.int.): 93(13), 41(28), 55(10), 67(10), 69(100), 81(62), 95(18), 121(12), 136(15), 137(13), 149(10). ^1^HNMR (CDCl_3_): 1.61 (9H, s, 3 × CH_3_), 1.69 (3H, s, CH_3_), 1.97 (6H, m), 2.09 (4H, m), 5.10 (1H, m), 5.14 (1H, m). HRMS: calcd. for C_15_H_24_ *m*/*z* 204.1878 (M^+^). Found: 204.1877. ^13^CNMR (CDCl_3_): 15.98 (q, CH_3_), 16.02 (q, CH_3_), 17.66 (q, CH_3_), 25.68 (q, CH_3_), 26.65 (t, CH_2_), 26.67 (t, CH_2_), 28.27 (t, CH_2_), 39.74 (tx2CH_2_), 124.26 (d, CH), 124.29 (d, CH), 124.40 (s, 4°C), 131.20 (s, 4°C), 134.84 (s, 4°C), 135.05 (s, 4°C). In this study, germacrene B is the first time to identify in BS.

### 3.2. Effects of BS on Vascular Permeability in Rats

**Figure 3:**
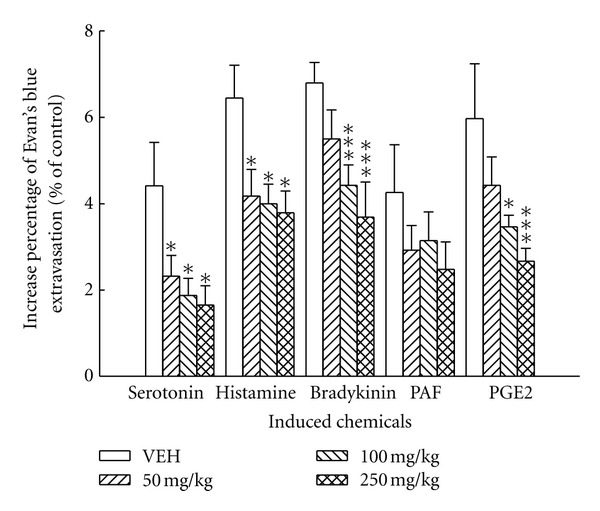
Effects of the crude extract of BS (50, 100, and 250 mg/kg) on the vascular permeability increased by serotonin, histamine, bradykinin, PAF, and PGE2 in rats. Each value represents the mean ± S.E. (*N* = 6). *P < 0.05, ***P < 0.001 as compared with the VEH (vehicle) group.

The abdominal Evan's blue extravasation in the marked circle intradermally with saline was represented as 100%. The increase percentage of the abdominal Evan's blue extravasations in the marked circle intradermally injected with inflammatory mediators was shown in Figure 3. It was shown that the BS crude extract (100, 250 mg/kg) reduced the extravasations induced by serotonin, histamine, bradykinin, and prostaglandin E_2_ but not PAF.

### 3.3. Anti-Inflammatory Activity of the BS Crude Extract, Various Layers of BS and Lupeol Acetate in Rats

**Figure 4:**
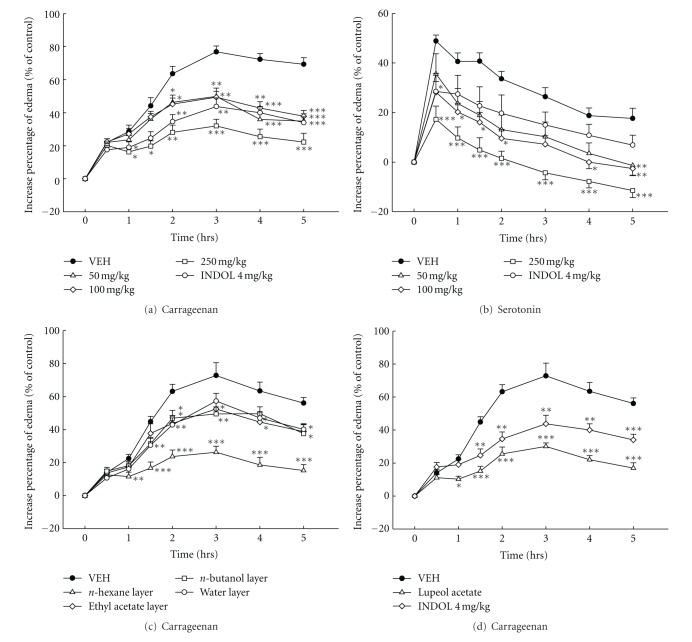
Effects of the crude extract, various layers of BS, lupeol acetate and indomethacin (INDOL, 4 mg/kg) on the carrageenan-induced (a, c, d) and serotonin-induced (b) paw edema in rats. The dosages of crude extract of BS were 50, 100, and 200 mg/kg. The dosage of various layers of BS and lupeol acetate was 100 mg/kg. Each value represents the mean ± S.E. (*N* = 6). *P < 0.05, **P < 0.01, ***P < 0.001 as compared with the VEH (vehicle) group.

As shown in Figure 4(a), the paw edema percentage caused by 1% carrageenan reached a maximum level (approximately 77.03%) at 3 hr. The crude extract of BS (50, 100, 250 mg/kg) notably decreased paw edema level from 1 hr to 5 hr after carrageenan administration. Indomethacin also decreased carrageenan-induced paw edema throughout the measurement intervals. The paw edema percentage caused by 1% serotonin reached a maximum level (about 48.52%) at 30 min. The BS crude extract (100, 250 mg/kg) decreased paw edema level significantly throughout the measurement intervals (Figure 4(b)). Indomethacin also decreased serotonin-induced paw edema only at 30 min after serotonin administration (*P* < 0.05). 

 Various layers of BS (100 mg/kg) decreased remarkably paw edema level from 1 hr to 5 hr after carrageenan administration (as shown in Figure 4(c)), and the inhibitory effect of *n*-hexane layer was better than the other layers. In Figure 4(d), lupeol acetate, isolated from *n*-hexane layer of BS, at 100 mg/kg also decreased significantly paw edema level from 1 hr to 5 hr after carrageenan administration. Indomethacin revealed a similar effect on carrageenan-indcued paw edema.

### 3.4. Antinociception Activity of the Crude Extract, Various Layers of BS and Lupeol Acetate in Mice

The inhibitory percentages and IC50 of antinociceptive activity of the crude extract, various layers of BS, and lupeol acetate were shown in Table 1. The BS crude extract, *n*-hexane, ethyl acetate, and water layers of BS (25 and 50 mg/kg) dose-dependently inhibited acetic acid-induced writhes. Lupeol acetate reduced acetic acid-induced writhes in a dose-dependent manner with inhibition of 45.8 and 83.7%, respectively. Acetylsalicylic acid and indomethacin decreased acetic acid-induced writhes with inhibition of 45% for both compounds. 

**Figure 5:**
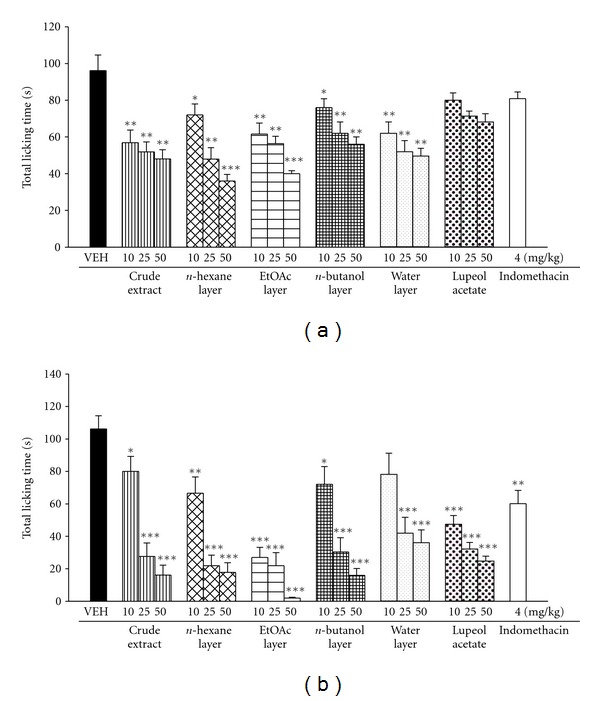
Effects of the crude extract, various layers of BS, lupeol acetate (10, 25, 50 mg/kg), and indomethacin (4 mg/kg) on early phase (0–5 min) (a) and late phase (10–35 min) (b) of formalin-induced licking response in mice. Each value represents the mean ± S.E. (*N* = 7). *P < 0.05, **P < 0.01, ***P < 0.001 as compared with VEH (vehicle) group.

In Figure 5, it showed the effects of BS crude extract, various layers of BS, and lupeol acetate (10, 25, 50 mg/kg) on the formalin-induced licking behavior in mice. BS crude extract at 10, 25, and 50 mg/kg decreased the licking time of the late phase induced by formalin in a dose-dependent manner. BS crude extract (50 mg/kg) inhibited the formalin-induced licking response by 50% in the early phase and by 85% in the late phase. The BS *n*-hexane, ethyl acetate, and water layers (25 and 50 mg/kg) also reduced the licking time of early and late phases in a dose-dependent manner (*P* < 0.001). Lupeol acetate significantly reduced the licking time of late phases in a dose-dependent manner (*P* < 0.001). Indomethacin (4 mg/kg) only inhibited the late but not early phase of formalin-induced licking in mice, with the maximal inhibition of 44% (*P* < 0.01).

### 3.5. Effects of Lupeol Acetate on LPS-Stimulated NO Production in RAW 264.7 Cells

**Figure 6:**
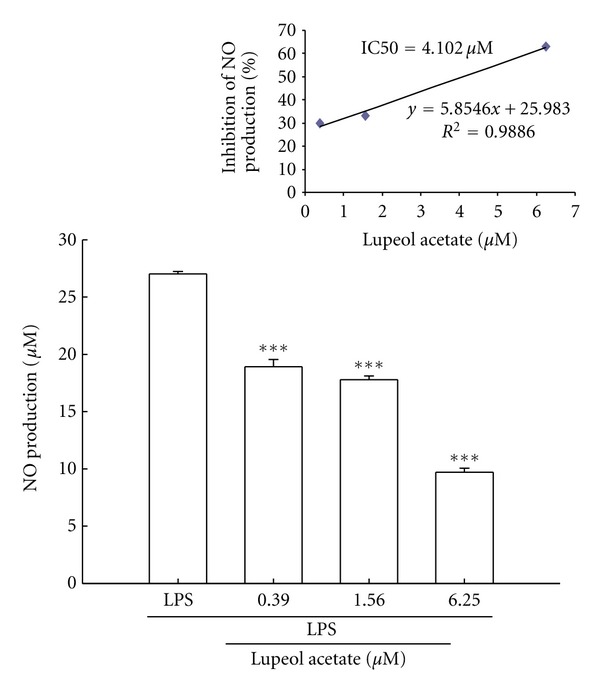
Effects of lupeol acetate on LPS-induced NO production in RAW 264.7 cells. Upper panel is the IC50 of inhibition of NO production, and lower panel is the NO production. Values represent mean ± S.E. of six separate experiments. ***P < 0.001 as compared with the LPS group.

LPS-induced NO production in RAW 264.7 cells was 27.02 *μ*M, and the inhibition of lupeol acetate (0.39, 1.56, 6.25 *μ*M) on NO production was 30%, 34%, and 64%, respectively. Lupeol acetate inhibited LPS-stimulated NO production in a dose-dependent manner (*P* < 0.001). The IC50 was 4.102 *μ*M (Figure 6).

### 3.6. Effects of Lupeol Acetate on LPS-Induced iNOS, COX2 Protein Expression in RAW 264.7 Cells

**Figure 7:**
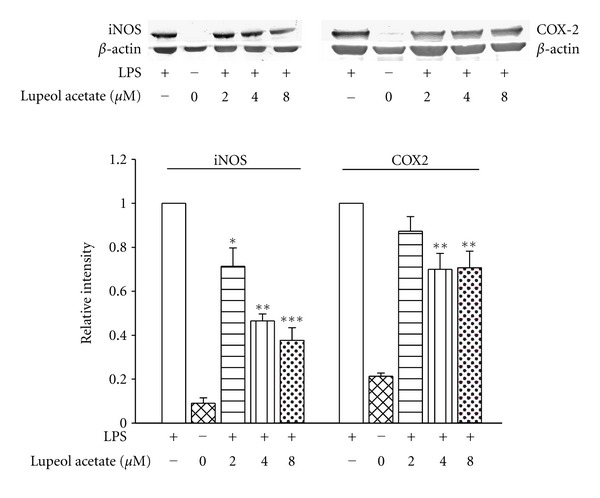
Effects of lupeol acetate on iNOS and COX-2 protein expression in LPS-stimulated RAW 264.7 cells. Protein expression (upper panel) and relative intensity (lower panel) of lupeol acetate treated group over LPS only treated group was measured using densitometer. *P < 0.05, **P < 0.01, ***P < 0.001 as compared with the LPS group.

Cells were treated with different concentration of lupeol acetate 2 hr prior to LPS treatement. Lupeol acetate (2, 4, 8 *μ*M) significantly reduced the iNOS protein levels by 28.29%, 53.46%, and 62.38%, respectively. Lupeol acetate (2, 4, 8 *μ*M) reduced LPS-induced COX-2 expression which was 12.67%, 30%, and 29%, respectively (Figure 7). The IC50 for lupeol acetate on iNOS and COX2 protein levels in LPS-stimulated RAW 264.7 cells was 5.35 *μ*M and 5.13 *μ*M, respectively. 

## 4. Discussion

Several inflammatory mediators such as autocrines, kinins, and prostaglandins, lead to a dilation of arterioles and venules and to increase vascular permeability, mediate edema formation and inflammation cascade [8, 9]. In addition, intradermal or subcutaneous injection of inflammatory mediators, such as lipopolysaccharide or lipoteichoic acid could be used as a model of inflammation. We examined the anti-inflammatory mechanism of the BS crude extract on plasma leakage in rats. BS crude extract decreased the Evan's blue extravasation induced by histamine, serotonin, bradykinin, and PGE2 but not PAF.

In order to further study the anti-inflammatory effect of BS, the chemical-induced edema formation was studied in rats. Carrageenan-induced edema usually is related to histamine, serotonin, kinins, PAF, and prostaglandins [10]. It was reported that carrageenan-induced edema usually separates into 3 phases. The first phase is 1.5 hrs after carrageenan treatment and is associated with the release of histamine, serotonin, and PAF. The second phase is 1.5 to 2.5 hrs after carrageenan treatment and is associated with kinins. The third phase, 2.5 to 5 hrs after carrageenan treatment is associated with prostaglandins and leukotrienes [14, 15, 19]. From the above results, it is demonstrated that the BS crude extract decreased carrageenan-induced edema throughout the measurement intervals in a dose-related manner and reached a maximal effect 3 hrs after carrageenan treatment. The effect of BS crude extract on carrageenan-induced paw edema is similar to indomethacin. Owing to serotonin is a proinflammatory mediator [20], the anti-inflammatory effect of BS crude extract on serotonin-induced edema was further assessed. The BS crude extract at higher dose (250 mg/kg) notably inhibited the serotonin-induced edema. The maximal effect of BS crude extract occurred approximately 30 min after administration. In addition, the inhibitory effect of *n*-hexane layer was better than the other layers of BS on the paw edema caused by 1% carrageenan. Lupeol acetate also decreased 1% carrageenan-induced paw edema. Results from inflammatory mediator-induced Evan's blue extravasation, or carrageenan- and serotonin-induced paw edema revealed that BS has anti-inflammatory effects partly via lupeol acetate acts on histamine, serotonin, and PGE_2_.

Formalin induced-licking behavior is a useful model for studying nociception and assessing analgesic drugs acting on central or peripheral effects. There are different physiological properties in the early and late phases of formalin-induced licking. The early phase is caused by central nerve fiber activation and the late phase is dependent on functional changes in peripheral nerves. In addition, Shibata et al. suggest bradykinin and substance P relate to the early phase of formalin-induced licking, and bradykinin, histamine, serotonin, and prostaglandin are associated with late phase of formalin-induced licking [12]. The crude extract and various layers of BS had both effects on the inflammatory algesia (late phase) and the neurogenic algesia (early phase). The IC50 of BS crude extract on early phase and late phase was 40.73 mg/kg and 23.67 mg/kg. The IC50 of *n*-hexane, ethyl acetate and water layers was 34.2 mg/kg, 37.21 mg/kg, and 46.24 mg/kg on the early phase and was 21.29 mg/kg, 13.17 mg/kg, and 30.39 mg/kg on the late phase, respectively. Lupeol acetate was more potent on the inflammatory algesia (late phase) than the neurogenic algesia (early phase). The IC50 of lupeol acetate on late phase of formalin test was 20.95 mg/kg. Moreover, the peripheral antinociceptive property of lupeol acetate was demonstrated from its inhibitory effect on acetic acid-induced writhing response in mice. 

NO may exhibit proinflammatory mediator [21], and produced by macrophages may contribute to systemic inflammatory diseases [22]. Therefore, inhibition of nitric oxide synthase (NOS) and NO production may be beneficial to ameliorate inflammatory diseases [22]. In this present study, lupeol acetate dose-dependently inhibited nitric oxide (NO) production, iNOS and COX-2 expression in LPS-stimulated macrophage RAW 264.7 cells. It is strongly revealed that the antinociceptive and anti-inflammatory effects of BS may be in a large part due to lupeol acetate.

In conclusion, BS has both antinociceptive and anti-inflammatory effects *in vivo* and *in vitro*. The mechanisms of BS may be related to modulation of the inflammatory mediators including autocrines, kinins, prostaglandins, and NO. Lupeol acetate inhibits NO production, iNOS and COX2 expression may take part in the antinociceptive and anti-inflammatory effects of BS. 

## Figures and Tables

**Table 1:** Effects of crude extract, each layer of *Balanophora spicata* and lupeol acetate (10, 25, and 50 mg/kg), acetylsalicylic acid (ASA, 150 mg/kg), and indomethacin (INDOL, 4 mg/kg) on the acetic acid-induced writhes in mice.

Groups	Dose (mg/kg)	Average writhing no.	Percentages of protection	IC50 (mg/kg)
VEH		27.89 ± 1.56	—	

Crude extract	10	21.43 ± 2.04	23.2	
	25	19.29 ± 1.82**	30.8	62.07
	50	17.36 ± 1.61***	37.8	

*n*-hexane layer	10	20.00 ± 1.04*	29.0	
	25	19.71 ± 1.06**	30.1	67.33
	50	17.91 ± 1.56***	36.5	

EtOAc layer	10	21.54 ± 1.88	23.6	
	25	18.70 ± 3.00**	33.7	56.81
	50	16.57 ± 3.66***	41.2	

*n*-butanol layer	10	24.32 ± 1.64	13.7	
	25	24.13 ± 2.51	14.4	91.36
	50	19.87 ± 3.60*	29.5	

Water layer	10	20.66 ± 0.93*	26.7	
	25	19.38 ± 2.84**	31.3	68.30
	50	18.21 ± 1.94**	35.4	

Lupeol acetate	10	21.04 ± 2.19	24.6	
	25	15.08 ± 2.46***	45.8	28.32
	50	4.56 ± 1.58***	83.7	

ASA	150	15.33 ± 0.78***	45	166.56

INDOL	4	15.45 ± 0.76***	45.2	4.484

Data are represented as mean ± S.E. *N* = 6 per group. *P < 0.05, **P < 0.01, ***P < 0.001 as compared with the VEH group.
